# Interpersonal Processes in the Duration of Sick Leave of Workers with Chronic Diseases: A Dyadic Analysis

**DOI:** 10.1007/s10926-024-10233-8

**Published:** 2024-09-03

**Authors:** Haitze J. de Vries, Nicole C. Snippen, Corné A. M. Roelen, Mariët Hagedoorn, Sandra Brouwer

**Affiliations:** 1 Department of Health Sciences, Community and Occupational Medicine, University of Groningen, University Medical Center Groningen, PO Box 196, 9700 AD Groningen, The Netherlands; 2 Arbo Unie, Nieuwegein, The Netherlands; 3 Department of Health Sciences, Health Psychology, University of Groningen, University Medical Center Groningen, Groningen, The Netherlands

**Keywords:** Dyadic processes, Illness perceptions, Return to work expectations, Significant others, Return to work, Chronic health conditions, Occupational health

## Abstract

**Purpose:**

Although there is increasing awareness that significant others’ perceptions and behavior can affect health outcomes, the role of interpersonal processes between sick-listed workers and significant others in sick leave and return to work (RTW) has hardly been studied. This study aims to examine the associations between illness perceptions, RTW expectations, and behaviors of significant others (engagement, buffering and overprotection) with sick leave duration within dyads of sick-listed workers with chronic diseases and their significant others.

**Methods:**

We used survey data linked with sick leave registry data of 90 dyads. Pearson correlations were used to study the interdependence within dyads. Multiple linear regression analyses were conducted to examine associations between survey data of both dyad members and sick leave duration.

**Results:**

We found moderate to strong correlations between workers and significant others, indicating interdependence within dyads regarding illness perceptions, RTW expectations and perceived significant other behaviors. Dyad members’ illness perceptions (*R*^*2*^ = .204, *p* = .001) and RTW expectations (*R*^*2*^ = .326, *p* =  < .001) were associated with sick leave duration, explaining respectively 12.3% and 24.5% of the variance. We found no associations between sick leave duration and active engagement, protective buffering and overprotection.

**Conclusions:**

This study indicates that negative illness perceptions and RTW expectations of both workers and their significant others are associated with a longer sick leave duration. Considering the interdependence within dyads, involving significant others when intervening on maladaptive illness perceptions and RTW expectations may be more effective than solely focusing on the worker’s perceptions and expectations.

**Supplementary Information:**

The online version contains supplementary material available at 10.1007/s10926-024-10233-8.

## Introduction

Many workers with chronic diseases experience difficulties coping with the consequences of their disease and are prone to negative work outcomes such as prolonged sickness absence [1] and early exit from paid employment [2] into unemployment [3] and disability pension [4]. Significant others like partners, family members and friends can play a key role in the coping and adaptation processes through their interactions with the person with the disease [5–7]. There is strong evidence that perceptions, coping and interactions within dyads can both positively and negatively affect behavioral, psychological, health and relationship outcomes [6–8]. For example, patient and partner perceptions, beliefs and expectations about an illness have been shown to be related to both one’s own and each other’s well-being and quality of life [6]. In addition, it has been shown that patients and significant others actively engaging in conversations about the situation and joint problem solving is associated with better outcomes like less distress and better relationship satisfaction [6, 7]. On the other hand, overprotectiveness and protective buffering (i.e., the efforts to deny or hide concerns or difficulties) of partners have been shown to be related to negative patient outcomes (e.g., decreased sense of control, more distress, worse physical well-being, lower adherence to medical advice) and worse relationship outcomes [6, 7].

In the context of work, previous studies have indicated that negative illness perceptions of workers concerning the duration, consequences, emotional impact, treatment efficacy, personal control and understanding of the illness are associated with increased risks of involuntary early labor market exit across various chronic health conditions [9–11]. Furthermore, return to work (RTW) expectations of workers have been shown to be one of the strongest prognostic factors of work outcomes like RTW, sick leave duration and work disability [11–14]. There is strong evidence that workers with low RTW expectations are at increased risk for long-term work disability [14] and that workers who expect a shorter sick leave duration have a higher probability of sustainable RTW [13].

Previous studies investigated the associations between illness perceptions, RTW expectations and work outcomes using a single perspective, focusing only on the worker. These studies fail to consider that cognitions like illness perceptions and RTW expectations and behaviors take place within a context in which the worker and significant other reciprocally influence each other [15, 16]. Only a few (mainly qualitative) studies have investigated the role of interpersonal processes between workers and their significant others in the RTW process. They suggest that negative illness perceptions and RTW expectations of significant others can hinder RTW as well [8, 17, 18], and that active engagement of significant others (e.g., sharing information, discussing RTW, listening to patients) can facilitate RTW, job retention and staying at work [8, 19–22]. In addition, protective or buffering behaviors from significant others (e.g., providing unnecessary assistance, encouraging or pressuring the worker to refrain from work, avoiding discussing the illness together) may negatively affect work outcomes [8, 19, 23].

To the best of our knowledge, our recently published cross-sectional study on dyadic associations between illness perceptions and RTW expectations [24] was the first quantitative study to use a dyadic design to investigate the role of interpersonal processes in the RTW process. The dyadic research design of this study allowed us to study both individual and interpersonal associations while taking the interdependence between dyad members into account [25]. We found that more negative illness perceptions of one member of the dyad were associated with more negative RTW expectations in both dyad members. For most illness perception domains, we found small to moderate actor and partner effects on RTW expectations. This study suggests that illness perceptions and RTW expectations should be considered at a dyadic level as workers and their significant others influence each other’s beliefs. However, from that study it remains unclear to what extent illness perceptions, RTW expectations and significant other behaviors within dyads are associated with sick leave duration.

Therefore, the aim of the present study was to examine whether illness perceptions, RTW expectations and significant other behaviors within dyads of sick-listed workers and their significant others are associated with sick leave duration of workers with chronic diseases. It was hypothesized that more negative illness perceptions and more negative RTW expectations of workers and their significant others are associated with a longer sick leave duration. With regard to significant other behaviors, we hypothesized that a higher level of active engagement reported by dyad members is related to a shorter sick leave duration, whereas higher levels of protective buffering and overprotection are associated with a longer sick leave duration.

## Methods

### Study Design and Procedure

This study used survey data of sick-listed workers and their significant others linked to sick leave register data with a maximum follow-up period of two years after the first day of sick leave. We included dyads consisting of workers who were on sick leave for at least two weeks due to a chronic health condition, and one of their significant others (i.e., partner, relative or friend). To be eligible for participation, workers had to be between 18 and 65 years of age, be on sick leave due to chronic health problems, and have a significant other (self-chosen by the worker) who was willing to participate in the study. They both had to be proficient in written Dutch.

The inclusion period lasted from June 2019 until September 2020. We recruited participants through local offices of a large Dutch occupational health service (OHS). For this study, an extra paragraph was added to the invitation for the consultation with an occupational health physician within six weeks after reporting sick, informing sick-listed workers and their significant others about the study. A link was included to a dedicated webpage with detailed information about the study and the online questionnaires. Both the worker and significant other were asked to individually complete the questionnaire.

At the start of the questionnaire, participants were screened for eligibility and asked to give informed consent for both using questionnaire results and retrieving OHS sickness absence register data. Participants who did not meet the inclusion criteria or did not give informed consent were excluded from participation.

### Measures

#### Sick Leave Duration

The outcome measure was the workers’ sick leave duration, which was defined as the time between the first day of reporting sick and the day of full RTW (i.e., at equal work hours as before sickness absence). We used register data with a maximum follow-up period of two years after the first day of sick leave. Workers who had not returned within that timeframe were assigned the maximum duration of 730 sick leave days. The outcome measure was based on sick leave register data of the OHS.

#### Illness Perceptions

Illness perceptions of workers and significant others were measured with respectively the Dutch version of the Brief Illness Perception Questionnaire (IPQ-B) [26, 27] and a significant other version of the IPQ-B, which was adapted from the spouse version of the IPQ-R [28]. In this study, the first eight items of the IPQ-B were used, which were measured on an ordinal response scale ranging from zero to ten. The items assessed the worker’s and significant other’s illness perceptions about: (1) the influence of the illness on the worker’s daily life (consequences), (2) the duration of the illness (illness duration), (3) the worker’s control over the illness (personal control), (4) the extent to which treatment can help with controlling the illness (treatment control), (5) the severity of the symptoms experienced by the worker (illness identity), (6) the worker’s concern about the illness (concern), (7) the worker’s emotional response to the illness (emotional response), and (8) the worker’s degree of understanding of the illness (illness coherence).

Higher scores on consequences, illness duration, illness identity, concern, and emotional response reflect more negative perceptions, while higher scores on personal control, treatment control, and illness coherence reflect more positive perceptions. A composite illness perceptions score was computed by summing the scores of the eight items, with a reverse scoring of the items on personal control, treatment control and illness coherence. For this composite score, we person-mean imputed data for participants with missing data on no more than three items. A higher composite score reflected more negative perceptions. In line with previous studies [29–31], the Cronbach's alpha of the IPQ-B composite score in this study was 0.71 for workers and 0.74 for significant others.

#### RTW Expectations

Expectations about the worker’s full RTW within 6 months were measured using a single-item measure, which has been demonstrated as a valid method to measure recovery expectations concerning RTW outcomes [32]. In this study, we used the ‘self-predicted certainty question’ [12]: “How certain are you that you will be fully back at work in six months?”. Workers answered the question on a 5-point scale: (1) “completely uncertain”; (2) “a little uncertain”; (3) “somewhat certain”; (4) “certain”; and (5) “completely certain”. Full RTW was defined as working the contracted working hours [12]. Significant others answered the question “How certain are you that the worker will be fully back at work in six months?” on the same 5-point scale.

#### Significant Other Behaviors

Significant other behaviors as perceived by the worker were measured with the Active engagement, Protective buffering, and Overprotection questionnaire (ABO) [33, 34]. The significant other version of the ABO was used to assess the self-perceived behaviors of significant others. The ABO contains 19 items measured on a 5-point scale ranging from "Never" to "Very often". Three subscales are distinguished: active engagement, protective buffering, and overprotection.

Active engagement was measured with 5 items, e.g. for workers “My significant other tries to discuss it with me openly”, and for significant others "I try to discuss it with the worker openly". Cronbach’s alpha was 0.88 for workers and 0.90 for significant others.

Protective buffering was measured with 8 items, e.g. for workers and significant others respectively “My significant other tries to hide his or her worries about me”, and “I try to hide my worries about the worker”. Because internal consistency was low for both workers and significant others (Cronbach’s alpha = 0.64 and 0.65), we deleted two items to improve this subscale. Cronbach’s alpha of the final subscale with 6 items was 0.74 for workers and 0.73 for significant others.

Overprotection was measured with 6 items, e.g., for workers and significant others respectively “My significant other continuously keeps an eye on me”, and “I continuously keep an eye on the worker”. The internal consistency of the scale with all six items was low (Cronbach’s alpha 0.65 for both workers and significant others). Two items were deleted from the scale. Cronbach’s alpha of the final subscale with 4 items was 0.80 for workers and 0.77 for significant others.

Supplementary Table 1 provides an overview of the items in each of the subscales and the deleted items.

#### Covariates

Socio-demographic characteristics and data about workers’ and significant others’ perceived relationship quality was collected to describe the sample and potentially include as covariates. Socio-demographic characteristics included the workers’ age (in years), gender, educational level (low, medium, or high), type of chronic disease (somatic, mental, mixed), and employment status (full-time vs. part-time). Likewise, data was collected about the significant others’ age, gender, educational level, chronic disease (yes/no), and their relationship with the worker (i.e., partner, parent, adult child, sibling, friend). Finally, we collected data from both workers and significant others about their perceived relationship quality with the other dyad member, using a relationship quality rating scale from 0–10, with zero representing the worst possible and ten the best possible relationship [35].

### Statistical Analyses

Pearson correlations were calculated to study the interdependence within dyads [25]; correlations of *r* = 0.1, *r* = 0.3, and *r* = 0.5 were considered to be weak, moderate and strong, respectively [36]. Following recommendations of Kenny on analyzing models with between-dyad outcomes [37], we conducted multiple linear regression analyses with the dyad as the unit of analysis to examine associations between survey data and sick leave duration. In preparation for the analyses, data was formatted in a dyadic structure with each row comprising one dyad. Preliminary analyses were performed to ensure that there was no violation of the assumptions of normality and homogeneity of variance. A series of preliminary analyses using one-way ANOVA and Pearson correlations was conducted to examine which socio-demographic and relationship measures should be controlled for as covariates in the analyses. Only the worker's gender (*F*(1, 88) = 4.91, *p* = 0.029) and full-time part-time employment status (*F*(1, 88) = 8.23, *p* = 0.005) were significantly associated with sick leave duration and therefore included as covariates in all analyses. Separate multiple regression models were tested for illness perceptions, RTW expectations, active engagement, protective buffering and overprotection. Furthermore, in the case of significant results, additional overall models were tested including respectively (i) illness perceptions and RTW expectations, and (ii) the three types of significant other behaviors. IBM SPSS version 26 was used to perform the analyses, applying a significance level of 0.05.

## Results

A total of *N* = 166 workers and *N* = 94 significant others completed the questionnaire. Due to the lack of data on the number of sick-listed workers and their significant others who received the invitation but chose not to participate, the response rate of participants is unknown. For the analyses, only cases with available survey data from both the worker and significant other and register data about sick leave were included. Therefore, workers for whom no survey data from a significant other (*n* = 72) or sick leave register data (*n* = 4) were available were excluded from the analyses. Consequently, the final study sample consisted of 90 workers and their significant others (dyads) (Table 1). There were no statistically significant differences between in- and excluded workers (Supplementary Table 2).

**Table 1:** Participant characteristics (*n* = 90 dyads)

Characteristic	Workers	Significant others
Age in years (*SD*)	53.5 (10.1)	52.5 (13.8)
*Gender*		
Male	49 (54.4%)	38 (42.2%)
Female	41 (45.6%)	52 (57.8%)
*Educational level*		
Low	16 (17.8%)	16 (18.0%)
Medium	31 (34.4%)	43 (47.8%)
High	42 (46.7%)	30 (33.3%)
Missing	1 (1.1%)	1 (1.1%)
*Relation to worker*		
Partner/spouse	–	81 (90.0%)
Parent	–	4 (4.4%)
Adult child	–	4 (4.4%)
Friend	–	1 (1.1%)
Relationship quality, mean (range)	8.7﻿ (6–10)	8.6 (5–10)
*Type of chronic disease*		
Somatic	56 (62.2%)	37 (39.4%)
Mental	17 (18.9%)	5 (5.3%)
Mixed	16 (17.8%)	6 (6.4%)
None	–	45 (47.9%)
Missing	1 (1.1%)	1 (1.1%)
*Number of chronic diseases*		
0	–	45 (47.9%)
1	51 (56.7%)	27 (28.7%)
> 1	38 (42.2%)	21 (22.3%)
Missing	1 (1.1%)	1 (1.1%)
*Employment status*		
Fulltime (≥ 36 h per week)	55 (61.1%)	24 (26.7%)
Part-time (12 – 35 h per week)	35 (38.9%)	37 (41.1%)
Not employed (< 12 h per week)	–	28 (26.7%)
Missing	–	1 (1.1%)
*Duration of sick leave, mean (range)*	323 (5–730)	
1 – 3 months	19 (21.1%)	
4 – 6 months	18 (20.0%)	
6 – 12 months	22 (24.4%)	
> 12 months	31 (34.4%)	
*Mean scores (SD)*		
RTW expectations (scale 1–5)	3.0 (1.3)	3.0 (1.4)
Composite illness perceptions score (scale 0–80)	48.5 (10.3)	46.1 (10.6)
Significant other active engagement (scale 1–5)	4.0 (0.8)	4.2 (0.7)
Significant other protective buffering (scale 1–5)	1.9 (0.6)	1.8 (0.6)
Significant other overprotection (scale 1–5)	1.4 (0.6)	1.4 (0.6)

The mean age of the workers was 53.5 years (SD = 10.1). About half of the workers was male (54.4%) and had a low or medium level of education (52.8%). Most workers (80.0%) indicated having a somatic disease, 36.7% of the workers had a mental illness, and almost half of the workers (42.2%) reported comorbid conditions. The mean age of significant others was 52.5 years (SD = 13.8), and the majority was the partner or spouse of the worker (90.0%).

To investigate the representativeness of the study sample, we compared our sample with a cohort retrieved from the same OHS, consisting of 3,729 workers with chronic diseases who were sick listed between January 2020 and September 2021. The mean age of workers was considerably higher in our study (53.7 years, SD = 9.9) than in the comparison cohort (40.4 years, SD = 15.9). Furthermore, compared to workers in that cohort, a higher percentage of workers in our study sample was male (54.4% vs. 33.6%), had a musculoskeletal disorder (47.9% vs. 34.5%) or a mental illness (36.2% vs. 24.4%).

### Interdependence within Dyads

The correlations between workers’ and significant others’ illness perceptions, RTW expectations, and perceived significant other behaviors are shown in Table 2. We found strong correlations between workers’ and significant others’ illness perceptions scores (*r* = 0.64), their expectations about the worker’s RTW (*r* = 0.80), and their perceptions of the significant other’s active engagement (*r *= 0.52). There were moderate correlations between workers’ and significant others’ perceptions of protective buffering (*r* = 0.46) and overprotection (*r* = 0.48) by significant others.

**Table 2:**
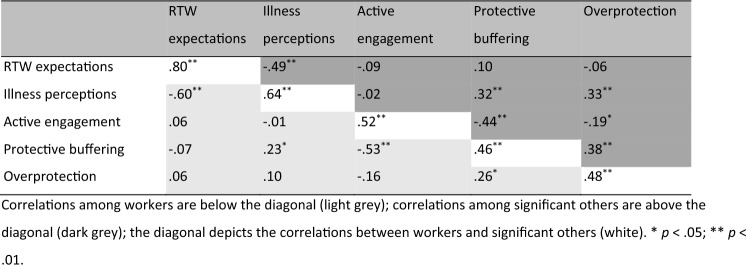
Intercorrelations of dyad members’ illness perceptions, return to work expectations, and significant other engagement, buffering and overprotection (condensed table)

### Associations with Sick Leave Duration

An overview of the multiple regression models is provided in Table 3.

**Table 3:** Results of the multiple linear regression models predicting duration of sick leave (adjusted for worker's gender and full-time/part-time employment)

	*B*	*95% CI*	*t*	*Sig*
**Model 1﻿: Illness perceptions (*n* = 90)**				
*Model 1a: Workers only*				
(Intercept)	124.15	[− 175.85, 423.85]	0.82	.412
Worker effect	8.58	[3.83, 12.34]	3.59	.001**
*Model 1b: Significant others only*				
(Intercept)	250.91	[− 41.00, 542.83]	1.71	.091
Significant other effect	6.46	[1.71, 11.22]	2.70	.008**
*Model 1c: Both dyad members*				
(Intercept)	96.29	[− 217.05, 409.64]	0.61	.543
Worker effect	7.32	[1.12, 13.53]	2.35	.021*
Significant other effect	1.91	[− 4.12, 7.94]	0.63	.530
**Model 2: Return to work expectations (*n* = 85)**				
*Model 2a: Workers only*				
(Intercept)	755.80	[555.90, 955.70]	7.52	< .001**
Worker effect	− 76.87	[− 113.78, − 39.96]	− 4.14	< .001**
*Model 2b: Significant others only*				
(Intercept)	840.29	[650.73, 1029.85]	8.81	< .001**
Significant other effect	− 92.47	[− 125.32, − 59.61]	− 5.60	< .001**
*Model 2c: Both dyad members*				
(Intercept)	840.62	[643.63, 1037.61]	8.49	< .001**
Worker effect	− 1.94	[− 60.61, 56.73]	− 0.07	.95
Significant other effect	− 90.95	[− 148.09, − 33.81]	− 3.17	.002**
**Model 3: Return to work expectations and illness perceptions (*n* = 85)**				
(Intercept)	701.94	[246.25, 1157.62]	3.07	.003**
Return to work expectations worker effect	4.13	[− 57.71, 65.98]	0.13	.895
Return to work expectations significant other effect	− 86.21	[− 145.43, − 26.98]	− 2.90	.005**
Illness perceptions worker effect	2.34	[− 4.28, 8.96]	0.70	.484
Illness perceptions significant other effect	− 0.21	[− 6.13, 5.71]	− 0.07	.943
**Model 4: Active engagement (*n* = 90)**				
*Model 4a: Workers only*				
(Intercept)	577.40	[277.71, 877.10]	3.83	< .001**
Worker effect	− 4.79	[− 76.06, 66.49]	− 0.13	.894
*Model 4b: Significant others only*				
(Intercept)	461.81	[114.01, 809.60]	2.64	.010*
Significant other effect	27.42	[− 52.78, 107.63]	0.68	.499
**Model 5: Protective buffering (*n*** ** =90)**				
*Model 5a: Workers only*				
(Intercept)	472.59	[153.83, 791.35]	2.95	.004**
Worker effect	31.89	[− 60.07, 123.84]	0.69	.492
*Model 5b: Significant others only*				
(Intercept)	716.95	[451.84, 982.06]	5.38	< .001**
Significant other effect	− 73.41	[− 162.81, 15.98]	− 1.62	.106
**Model 6: Overprotection (*n*** **= 90)**				
*Model 6a: Workers only*				
(Intercept)	596.66	[361.98, 831.33]	5.05	< .001**
Worker effect	− 24.00	[− 120.12, 72.13]	− 0.50	.621
*Model 6b: Significant others only*				
(Intercept)	618.40	[390.60, 846.21]	5.40	< .001**
Significant other effect	− 38.19	[− 124.98, 48.59]	− 0.88	.384

^*^p < .05 **p < .01

#### Model 1: Associations of Illness Perceptions with Sick Leave Duration

When entered separately in regression models, the illness perceptions of both workers (*B* = 8.58, *p* = 0.001) and significant others (*B* = 6.46, *p* = 0.008) were significantly associated with the worker’s sick leave duration, with more negative illness perceptions being associated with a longer sick leave duration.

The regression model 1c, including covariates and the illness perceptions of both dyad members, was statistically significant (*F(4, 85)* = *5.44, R*^*2*^ = 0.204, *p* = 0.001). The illness perceptions of workers and significant others explained 12.3% of the variation in sick leave duration. When including dyad members’ illness perceptions simultaneously, only the coefficient associated with the worker’s illness perceptions remained statistically significant (*B* = 7.32, *p* = 0.021).

#### Model 2: Associations of RTW Expectations with Sick Leave Duration

When entered separately in regression models, the RTW expectations of workers (*B* = −76.87, *p* < 0.001) and significant others (*B* = −92.47, *p* < 0.001) were both significantly associated with sick leave duration. More positive RTW expectations of dyad members were associated with a shorter sick leave duration.

The regression model 2c, including covariates and the RTW expectations of both dyad members, was statistically significant (*F*(4, 80) = 9.66, *R*^*2*^ = 0.326, *p* =  < 0.001). The RTW expectations of workers and significant others accounted for 24.5% of the variance in sick leave duration. When including dyad members’ RTW expectations simultaneously, only the coefficient associated with the significant other’s RTW expectations remained significant (*B* = −90.95, *p* = 0.002).

#### Model 3: Associations of Illness Perceptions and RTW Expectations with Sick Leave Duration

The regression model including covariates, both dyad members’ illness perceptions and their RTW expectations was statistically significant (*F*(6, 78) = 6.42, *R*^*2*^ = 0.331, *p* =  < 0.001). Illness perceptions and RTW expectations accounted for 25.0% of the variation in sick leave duration. In this model, only the coefficient associated with RTW expectations of the significant other significantly contributed to the model (*B* = −86.21, *p* = 0.005).

#### Models 4, 5, and 6: Associations of Significant Other Behaviors with Sick Leave Duration

There were no significant associations with sick leave duration for workers’ and significant others’ perceptions about the significant other’s active engagement, protective buffering and overprotection. As the assumption of normality for the variable overprotection was violated among both workers and significant others, we performed sensitivity analyses with median-split dummy variables. No significant associations were found. Because no significant associations with sick leave duration were found for dyad members’ perceptions about the significant other’s behaviors, no multiple linear regressions were performed including both dyad members’ perceptions simultaneously.

## Discussion

The findings of this study add to our understanding of the role of interpersonal processes in sick leave duration of workers with chronic diseases. The moderate to strong correlations between workers and significant others found in this study indicate that dyad members’ illness perceptions, RTW expectations and perceptions about significant other behaviors are interdependent. More negative illness perceptions and more negative RTW expectations of both dyad members were associated with a longer sick leave duration. No significant associations were found between workers’ and significant others’ perceptions about the significant other’s active engagement, protective buffering and overprotection and sick leave duration.

The findings on the associations of illness perceptions and RTW expectations with sick leave duration confirm that interpersonal processes within dyads play a role in sick leave duration of workers with chronic diseases. The strong correlations within dyads suggest that workers and their significant others may develop shared illness perceptions and RTW expectations. While this finding may not be unexpected given that dyad members likely shared information about the illness and RTW prognosis, previous research indicates that differing perceptions within dyads are not uncommon [38, 39]. In line with our hypotheses, we found that more negative illness perceptions and RTW expectations within dyads are related to a longer sick leave duration. These findings are in line with previous studies, which showed that workers’ illness perceptions and RTW expectations affect work participation, sick leave and RTW [9–14]. In addition, our results confirm findings from the prior qualitative studies that negative illness perceptions and RTW expectations of significant others can hinder RTW [8, 17, 18].

When including independent variables of both dyad members, we found that in the illness perceptions model only the coefficient of workers (i.e., actor effect) remained significant, whereas in the RTW expectations model only the coefficient of significant others (i.e., partner effect) remained significant. While it is possible that one dyad member’s perceptions or expectations has a stronger influence on sick leave duration than those of the other dyad member, the rather high correlations within dyads could also have resulted in either a significant actor effect or partner effect by chance while in fact they do not differ significantly.

In contrast to our hypotheses and previous research [40], no significant associations were found between dyad members’ perceptions about the significant other behaviors (i.e., active engagement, protective buffering, and overprotection) and sick leave duration. A possible explanation for this is the distribution of scores on active engagement, protective buffering, and overprotection among participating workers and significant others. Specifically, the majority of participants reported high levels of active engagement and low levels of protective buffering and overprotection. Only three to ten percent of the participants scored below three on active engagement or above three on protective buffering and overprotection. Consequently, the statistical power to detect an association between these significant other behaviors and duration of sick leave was likely reduced. Furthermore, we used generic measures of significant other behaviors, which may not fully capture work-related responses that can influence sick leave duration. This may also in part explain why we did not find any evidence that significant other behaviors are associated with duration of sick leave, whereas a prior study using specific measures regarding family members’ attitude to RTW and support for RTW did find significant associations with RTW outcomes [40]. As context specific measures have been found to be more sensitive for the detection of associations and effects than generic measures [41], it is likely that measures specifically on work-related responses from significant others are more predictive of sick leave duration than generic measures as used in this study.

### Strengths and Limitations

This study has several strengths and limitations. First, the dyadic design allowed us to study both individual and interpersonal associations while taking the dyad members’ interdependence into account. As such, in this study we explicitly acknowledge that illness perceptions, RTW expectations and responses take place within an interpersonal context in which dyad members reciprocally influence each other. Other strengths are the use of validated patient- and significant other-versions of the questionnaires to measure illness perceptions and perceived significant other behaviors. Moreover, sick leave duration as outcome measure was based on register data, which restricts recall bias.

A limitation of this study is that the study sample included more workers of older age, more men and workers with a musculoskeletal- or mental condition in comparison to a representative cohort of the OHS. This selection bias may have influenced our results in case dyadic processes differ depending on age, gender, or type of disease. However, we do not expect this to be the case, as preliminary analyses indicated that it was not necessary to control for age or type of chronic disease and we included both dyad members’ gender as covariates. Second, our findings indicate the presence of nonresponse bias, with an overrepresentation of workers and significant others who were highly satisfied with their relationship, and who reported high levels of active engagement and low levels of protective buffering and overprotection by the significant other. Nonresponse bias is not uncommon, and it is a known phenomenon that individuals who are more satisfied with their relationship are often overrepresented in dyadic study samples [42–44]. Therefore, while the findings of this study apply to dyads who are highly satisfied with their relationship and in which significant others exhibit high levels of active engagement and low levels of protective buffering and overprotection, they may not generalize to dyads that are less satisfied with their relationship. Further research is needed to investigate whether the present results reproduce in other study populations and whether the interpersonal associations between illness perceptions, RTW expectations and sick leave duration differ depending on the worker’s disease and relationship satisfaction of dyad members. Another limitation of this study is that we used generic measures of significant other behaviors rather than an instrument designed to measure work-specific responses that are likely more predictive of sick leave duration. However, to our knowledge, no such validated instruments currently exist. Due to constraints in time and resources, we decided to use the validated ABO questionnaire to assess significant others’ behaviors. A critical direction for future research is the development and validation of an instrument tailored to measure work-related responses from significant others. Such an instrument could assess factors like support in executing re-integration plans and behaviors that encourage or discourage RTW.

### Practical Implications

This study shows that illness perceptions and RTW expectations of workers and their significant others are interdependent and associated with sick leave duration of workers with chronic diseases. Assessing these factors can not only help occupational health professionals to identify workers at higher risk of long-term sickness absence, but can also provide insight into inadequate or maladaptive perceptions and expectations among workers and significant others that may be modified to facilitate return to work [45]. In this context, occupational health professionals can explore RTW expectations by asking workers and significant others about their thoughts about the worker’s ability to return to work and when they expect the worker to be back at work [32]. Furthermore, illness perceptions of workers and significant others could be explored by asking about their views of the worker’s illness, or with the revised or brief version of the Illness Perception Questionnaire (IPQ) [46–48].

Considering the interdependence within dyads of workers and significant others, dyadic approaches in RTW processes could be of particular importance when dyad members’ illness perceptions or RTW expectations are inadequate or maladaptive. In that case, involving significant others in efforts to promote adaptive illness perceptions and RTW expectations may be more effective than an individualistic approach that solely targets the worker’s perceptions and expectations. Consequently, occupational health professionals should consider adopting a dyadic approach to modify illness perceptions and RTW expectations that hinder recovery and sustainable return to work. In this context, occupational health physicians can facilitate accurate and adaptive illness perceptions and RTW expectations of workers as well as their significant others by providing information about the worker’s disease and the RTW process [46, 48–51]. Moreover, informing significant others about the re-integration plans and actively involving them in the decision-making process can help in managing their expectations about recovery and RTW. This approach can also support workers and significant others in adopting more effective coping strategies.

## Conclusion

The findings of this study indicate that illness perceptions and RTW expectations of workers and their significant others are interdependent and associated with sick leave duration of workers with chronic disease. More negative illness perceptions and more negative RTW expectations of both workers and their significant others were found to be associated with a longer sick leave duration. Perceived active engagement, protective buffering and overprotection by significant others were not associated with workers’ sick leave duration. Considering the interdependence within dyads, involving significant others when intervening on maladaptive or inadequate illness perceptions and RTW expectations may be more effective than targeting only the worker’s perceptions and expectations.

## Supplementary Information

Below is the link to the electronic supplementary material.

Supplementary file1 (DOCX 21 kb)

## Data Availability

The dataset generated and/or analyzed during the current study is not publicly available, but is available from the corresponding author upon reasonable request.

## Abbreviations

RTW: Return to work
OHS: Occupational health services
IPQ-B: Brief illness perception questionnaire
ABO: Active engagement, protective buffering, and overprotection questionnaire
