# Supplementary material for: Interpersonal Processes in the Duration of Sick Leave of Workers with Chronic Diseases: A Dyadic Analysis
Source: J Occup Rehabil. 2024 Sep 3;35(3):654–64. doi: 10.1007/s10926-024-10233-8 (PMC12361268; doi:10.1007/s10926-024-10233-8)
Supplement: Supplementary file 1 — Supplementary file1 (DOCX 21 kb) [file 10926_2024_10233_MOESM1_ESM.docx]

Supplementary tables

**Supplementary Table 1.** Overview of items included and deleted items for the significant other behavior scales

|  | **Original scale** | | | | **Final scale** | | | |
| --- | --- | --- | --- | --- | --- | --- | --- | --- |
|  | *Workers* | | *Significant others* | | *Workers* | | *Significant others* | |
| **Items** | *α if item deleted* | *α* | *α if item deleted* | *α* | *α if item deleted* | *α* | *α if item deleted* | *α* |
| Active engagement |  | .88 |  | .90 |  |  |  |  |
| **My partner tries to discuss it [the fact that I am ill] with me openly** | .84 |  | .88 |  |  |  |  |  |
| **My partner asks me how I feel.** | .85 |  | .89 |  |  |  |  |  |
| **When something bothers me, my partner tries to discuss the problem.** | .84 |  | .85 |  |  |  |  |  |
| **My partner is full of understanding towards me.** | .87 |  | .88 |  |  |  |  |  |
| **My partner makes me feel that I'm not alone in this.** | .88 |  | .88 |  |  |  |  |  |
| Protective buffering |  | .65 |  | .64 |  | .74 |  | .73 |
| **With the best intentions, my partner makes up excuses to persuade me to follow the doctor's instructions.** | .66 |  | .60 |  | .79 |  | .72 |  |
| **My partner tries to hide his or her worries about me.** | .58 |  | .60 |  | .70 |  | .74 |  |
| **My partner tries to act as if nothing is the matter.** | .56 |  | .57 |  | .65 |  | .66 |  |
| **My partner gives in when I make an issue of something.** | .61 |  | .56 |  | .74 |  | .67 |  |
| **My partner just waves my worries aside.** | .59 |  | .59 |  | .68 |  | .68 |  |
| My partner does everything to prevent me from thinking about my disease. | .68 |  | .69 |  | Deleted |  | Deleted |  |
| **My partner can't endure me being concerned and acts as if he or she doesn't notice my worries.** | .53 |  | .57 |  | .64 |  | .68 |  |
| My partner takes over as much of my work as possible. | .68 |  | .63 |  | Deleted |  | Deleted |  |
| Overprotection |  | .65 |  | .65 |  | .80 |  | .77 |
| **My partner treats me like a baby.** | .62 |  | .61 |  | .79 |  | .80 |  |
| My partner continuously keeps an eye on me. | .63 |  | .64 |  | Deleted |  | Deleted |  |
| My partner takes care that I follow the doctor's instructions. | .70 |  | .66 |  | Deleted |  | Deleted |  |
| **When it comes down to it, my partner seems to think that he or she can't leave my recovery to me.** | .55 |  | .60 |  | .69 |  | .68 |  |
| **When it comes down to it, my partner seems to think that when he or she is not constantly around, I will not follow the doctor's instructions.** | .56 |  | .57 |  | .77 |  | .68 |  |
| **When it comes down to it, my partner seems to think that I don't know what's right for me.** | .58 |  | .58 |  | .71 |  | .67 |  |
| Bold items were included in the final scale |  |  |  |  |  |  |  |  |

**Supplementary Table 2.** Comparison baseline characteristics of in- and excluded workers

| **Characteristic** | **Included workers (n = 90)** | | **Excluded workers (n = 76)** | | **Sig** |
| --- | --- | --- | --- | --- | --- |
| Age in years (SD) | 53.5 | (10.1) | 52.5 | (14.4) | .605 |
| Gender |  |  |  |  | .475 |
| Male | 49 | (54.4%) | 44 | (57.9%) |  |
| Female | 41 | (45.6%) | 31 | (40.8%) |  |
| Educational level |  |  |  |  | .235 |
| Low | 16 | (17.8%) | 16 | (21.1%) |  |
| Medium | 31 | (34.4%) | 34 | (44.7%) |  |
| High | 42 | (46.7%) | 26 | (34.2%) |  |
| Relationship quality, mean (SD) | 8.7 | (1.0) | 8.3 | (1.9) | .075 |
| Type of chronic disease |  |  |  |  | .634 |
| Somatic | 56 | (62.2%) | 40 | (52.6%) |  |
| Mental | 17 | (18.9%) | 16 | (21.1%) |  |
| Mixed | 16 | (17.8%) | 16 | (21.1%) |  |
| Number of chronic diseases |  |  |  |  | .461 |
| 1 | 51 | (56.7%) | 46 | (60.5%) |  |
| >1 | 38 | (42.2%) | 27 | (35.5%) |  |
| Employment status |  |  |  |  | .373 |
| Fulltime (≥ 36 hours per week) | 55 | (61.1%) | 39 | (43.4%) |  |
| Part-time (12 – 35 hours per week) | 35 | (38.9%) | 33 | (51.3%) |  |
| Duration of sick leave (max 730 days), mean (SD) | 323 | (254) | 282 | (216) | .314 |
| Mean scores (SD) |  |  |  |  |  |
| RTW expectations (scale 1-6) | 3.0 | (1.3) | 3.1 | (1.3) | .780 |
| Composite illness perceptions score (scale 0-80) | 48.5 | (10.3) | 47.2 | (11.4) | .374 |
| Significant other active engagement (scale 1-5) | 4.0 | (0.8) | 3.9 | (0.7) | .961 |
| Significant other protective buffering (scale 1-5) | 1.9 | (0.6) | 2.0 | (0.6) | .708 |
| Significant other overprotection (scale 1-5) | 1.4 | (0.6) | 1.6 | (0.6) | .190 |
